# The Mediating Role of Migrant Community-Based Organizations: Challenges and Coping Strategies

**DOI:** 10.1007/s11266-023-00572-0

**Published:** 2023-05-04

**Authors:** Sara Martinez-Damia, Daniela Marzana, Virginia Paloma, Elena Marta

**Affiliations:** 1 Università Cattolica del Sacro Cuore, Largo Gemelli 1, 20123 Milan, Italy; 2 Universidad de Sevilla, Sevilla, Spain

**Keywords:** Community participation, Immigrants, Mediating structures, Migrant community-based organizations, Social justice

## Abstract

Migrant community-based organizations (MCBOs) are key mediating structures between immigrants and host societies. However, when implementing this role in host societies, MCBOs often face a number of challenges that reduce their chances to be effective in promoting social justice. This paper aims to analyze the challenges that MCBOs settled in Milan (Northern Italy) experience and the coping strategies that they use in order to provide some guidelines on how to support them. In-depth interviews, observations and document analysis with 15 MCBOs were conducted. Based on a situational analysis, we present the main challenges perceived by MCBOs at three levels: internal (i.e., surviving), inter-organizational (i.e., collaborating) and community (i.e., being recognized as mediating actors). We provide specific guidelines for action on how to address such challenges and thus foster the role of MCBOs as mediating structures in receiving societies.

## Introduction

Migration has been defined as the “act of leaving one’s place of birth and moving across national borders in pursuit of greater capacity to self-determine through territorial admission and political inclusion” (Achiume, 2019, p. 1552). In EU Members States, except for certain categories such as refugees, immigrants are kept outside of the political borders that states created. For the immigrants who are able to enter, arrival is characterized by many barriers to self-determination capacity, and expectations on higher life satisfaction in host societies are not always met (Hendriks, 2015). Institutional discrimination and anti-immigrant prejudice as well as negative media narratives are an everyday reality for immigrants (Eberl et al., 2018; Eurobarometer, 2019) with a harmful impact on various aspects of their health (Jasinskaja‐Lahti et al., 2006; King & Mai, 2012; Lajevardi et al., 2020; Safi, 2010) and on society as a whole (Esses, 2021).

In the last years, several authors (Bloemraad & Terriquez, 2016; Buckingham et al., 2021; Guo, 2014) have highlighted the meaningful contribution migrant community-based organizations (MCBOs) have in helping immigrants face the hardship of migration. According to the liberation community psychology approach in societies, there are oppressed groups with limited resources for decent living because of historical, cultural and social forces (Garcia-Ramirez et al., 2009; Martín-Baró, 1986; Paloma & Manzano-Arrondo, 2011). According to this approach, immigrants in host societies cannot thrive and may self-identify with denigrating stereotypes. Nevertheless, they can try to transform their situation by organizing and actively participating in the society. Their liberation represents an empowering, democratic and ethical–critical process that can occur within MCBOs.

Community-based organizations can be seen as mediating structures for the central role they play in building change both in the individual and social spheres and in facilitating relationships between immigrants and locals (Berger & Neuhaus, 1977; Todd & Allen, 2011). The European Commission (2020) highlighted the importance of creating spaces for immigrants and host communities to meet, providing the former with support for their long-term integration and the latter with an opportunity to learn about people entering their communities. MCBOs are precisely these spaces.

Against this background, it is important to identify what challenges and coping strategies these organizations have and find ways to support them in their endeavors. However, the lack of up-to-date research in this area reduces the possibility for MCBOs to effectively play their mediating role and become agents of change within host societies (Maton, 2008). Using situational analysis, this paper aims to analyze the main challenges and coping strategies that MCBOs settled in Northern Italy experience and how these challenges were experienced according to some organization-specific markers.

### The Mediating Role of Migrant Community-Based Organizations within Host Societies

MCBOs are organizations founded by immigrants to serve their own community and where immigrants have a significant presence on the board of directors (Babis, 2016). According to the liberation psychology approach, community-based organizations can act as catalyst for the transformation of immigrants’ psychological patterns of internalized oppression and of oppressive social structures (Martin-Barò, 1994; Paloma & Manzano-Arrondo, 2011). MCBOs can function as mediating structures as they situate themselves in an “ideal area” (Paloma & Manzano-Arrondo, 2011, p. 312), namely between the structural and individual level where changes required to bring social justice and individual well-being can be synergistically promoted (Prilleltensky & Prilleltensky, 2006). Mediating structures are those that “provide unique contexts that may influence, or ‘mediate,’ how individuals engage with society and social problems, and often serve as the practical bridges between individuals and society” (Todd & Allen, 2010, p. 222).

On the one hand, many scholars reported that MCBOs increase cultural capital, sense of community and multiculturalism in the receiving societies (Bloemraad & Terriquez, 2016; Guo, 2014; Handy & Greenspan, 2009). MCBOs can also “have benefits such as […] counteracting demeaning and exclusionary narratives” (Gigliotto et al., 2022, p. 85). They can be empowering community settings as they can influence community enhancement and social change (Paloma et al., 2010). On the other hand, individuals may use MCBOs as a vehicle for their own well-being. Indeed, MCBOs usually provide material (e.g., information, money, legal advice) and immaterial support (e.g., psychological assistance, trustworthy relations, encouragement) to newly arrived migrants who can share concerns and network and fight for their rights in the receiving societies (Aceros et al., 2021; Bonnett, 1977; Marzana et al., 2020a, 2020b; Paloma et al., 2010). They can also function as settings where immigrants’ cultural identities are protected and differences are negotiated (Martinez-Damia et al., 2021; Marzana et al., 2019; Sonn, 2002; Taurini et al., 2017). In line with this, research found that immigrants who participate within community-based organizations are more integrated and have higher psychological well-being compared with those who do not participate (Martinez-Damia et al., 2023; Marzana et al., 2020a, 2020b). Nevertheless, the liberation of immigrants from oppressive contexts depends on the challenges that MCBOs face when playing their mediating role. Therefore, the benefits that immigrants can gain from participating in these settings—as well as in the host society as a whole—are rooted in MCBOs’ ability to promote social justice.

### Challenges of Migrant Community-Based Organizations in Acting as Mediating Structures

Some authors reported that MCBOs may not be effective in their mediating role of promoting social justice due to a lack of power and ability to advocate for immigrants’ rights in host societies (Paloma & Manzano-Arrondo, 2011). Indeed, MCBOs are often ignored and excluded from negotiations with public institutions for several reasons (Kyrieri & Brasser, 2012; Papadopoulos et al., 2012; Sezgin, 2008) and hence may perpetuate the status quo instead of fighting it.

Firstly, the type and quality of relationships and contacts that MCBOs are able to establish with others social actors may not be appropriate (Gigliotto et al., 2022; Kyrieri & Brasser, 2012; Ortiz, 1981). Scholars have identified phenomena such as inferiorization, manipulation and culturalization (Gigliotto et al., 2022; Kyrieri & Brasser, 2012). Inferiorization refers to MCBOs not feeling fully acknowledged and taken seriously by national organizations, while manipulation occurs when MCBOs are engaged in projects only to recruit immigrants without participating in the design and management phases. Culturalization refers to “the imposition of certain culturally—or professionally—based practices that may not be shared by the participants” (Gigliotto et al., 2022, p. 83). Considering that MCBOs get some of their resources through the connections they have with national organizations (Pilati, 2012), these phenomena represent a disadvantage for MCBOs as they see their power compromised. Their power is also impaired as they have a lower level of social capital and resources compared with other non-profit national organizations (Gleeson & Bloermraad, 2013; Papadopoulos et al., 2013).

Secondly, opportunities for social justice may be less for MCBOs as they usually have weak organizational structure. Indeed, MCBOs mostly rely on volunteers who have less time for their activities and are more oriented to solve the everyday problems of immigrants (Papadopoulos et al., 2013). MCBOs have also less opportunities to access venues as they face the hurdles of applying for funding (Gigliotto et al., 2022). This helps to understand why MCBOs experience higher instability in their activities (Kyrieri & Brasser, 2012).

While some steps have been made to explain the hardships MCBOs face in playing their mediating role in promoting social justice, research is not up-to-date and we still do not know a lot on the coping strategies and the differences experienced according to the features of these organizations. Are MCBOs of immigrants from South America experiencing the same challenges of MCBOs of immigrants from Africa or Eastern Europe? Are there differences between MCBOs with a long history and those founded more recently? Are there different challenges based on the activities that MCBOs do? More studies are required. This research aims to (a) analyze the main challenges and coping strategies that MCBOs settled in Northern Italy experience and (b) identify how these challenges are experienced according to some organization-specific markers (i.e., geographical area of origin, year of foundation, area of interest).

## Methods

### Study Context

Italy is an example of how European societies address migration. In the country, media propaganda has framed migration as a security issue for over nearly two decades (Mazzara et al., 2020) and as a public-order problem that threatens Italians (Barretta, 2019). Historical research on Italian migration policies shows that, in Italy, immigrants faced a policy that excluded them on multiple levels through laws that were based on flow regulation and emergency (Ambrosini, 2013; Caneva, 2014). At the time of data collection, two decrees on security and immigration with negative effects on the well-being of immigrants were approved.

In Italy, there are 1149 MCBOs and Lombardy—situated in the North of the country—is one of the regions with the most MCBOs, 59.9% of which are in the city of Milan (Centro Studi e Ricerche IDOS, 2021). In Italy, small–medium MCBOs are usually run by volunteers, do not have paid staff and are funded by member donations. Immigrants need to have legal status in the country to establish a MCBO. There are mainly two juridical forms for MCBOs: APS (Associazioni di Promozione Sociale, Social Development Organizations), where members promote social and cultural activities among themselves, and ODV (Organizzazioni di Volontariato, Volunteer Organizations), where members run activities to help someone else. After choosing the legal form of the organization, immigrants need to establish the aims of the organizations, define a statute and pay some registration fees to the state. Over the years, MCBOs in the Milan area were able to establish connections with immigration services such as prefectures, police headquarters, health and legal organizations, and consulates.

The present study is part of a larger multi-method project [https://www.partecipazioneimmigrati.it/] that aims to analyze the organizational challenges and individual benefits of community participation among immigrants in Italy. In this article, we present the results related to the proposed research questions.

### Participants and Sampling Strategy

Our recruitment of MCBOs started with an umbrella organization called Associazione Città Mondo (ACM). ACM was established during the 2015 EXPO in Milan to bring together all migration-related organizations. Within ACM, there are Italian non-profit organizations, businesses and MCBOs. We started our fieldwork in July 2019 by selecting from the ACM database the organizations that had been founded by immigrants and still had immigrants within the board of directors. A database was created with 33 MCBOs that were categorized based on organization-specific markers: (a) year of foundation of the MCBO—an indicator of how many years the MCBO had been active; (b) geographical area of origin of members; (c) area of interest where activities were conducted, such as social service (e.g., language courses, legal and health assistance), cultural service (e.g., preservation of cultural heritage and traditions though arts) and advocacy (e.g., campaigns for immigrants’ rights).

We contacted the identified MCBOs to outline the research. Eleven MCBOs could not be reached as they did not answer emails or phone calls, five did not want to take part in the research, eight were excluded because they were not constantly active and nine were interested in the research. We reached six more MCBOs through both the snowballing technique (i.e., new MCBOs were reported by those who had already been involved) and a new field research on MCBOs active in the city. The majority of MCBOs did not have Italians as their volunteers and used Facebook as main communication channel.

Fifteen MCBOs were involved in the research. All MCBOs were medium–small community-based organizations (i.e., the number of active volunteers ranged from 1 to 15), were both APS and ODV and had members coming from developing countries. In our sample, the majority of MCBOs had been established between 2000 and 2010, have members who came from different geographical areas and were involved mainly in social service activities. Table 1 shows the characteristics of MCBOs that participated in the study.

**Table 1:** Characteristics of migrant community-based organizations included in the study

MCBO	Data source	Foundation	Geographic area of origin	Area of interest
1	1 Interview (3/5/20), 1 Observation (11/16/19)	Historical	South America	SS and A
2	2 Interviews (3/6/20; 6/13/20), Statute	Historical	West Africa	SS
3	1 Interview (3/5/20), Statute	Recent	Eastern Europe	CS and SS
4	3 Interviews (1/23/20; 6/24/20; 6/25/20), 2 Observations (11/7/19; 1/16/20)	Recent	South America	SS and A
5	2 Interviews (2/12/20; 6/17/20), Statute	Recent	South America	SS
6	1 Interview (2/13/20), 1 Observation (12/8/19), Statute	Recent	Eastern Europe	SS
7	3 Interviews (2/14/20; 2/28/20; 3/10/20)	Recent	South America	SS
8	1 Interview (6/24/20)	Recent	Asia	SS
9	2 Interviews (6/22/20; 7/15/20), Statute	Recent	Asia	CS and SS
10	1 Interview (3/6/20), Statute	New	Eastern Europe	CS
11	2 Interviews (3/7/20; 3/10/20), Statute	New	Eastern Europe	CS
12	2 Interviews (6/11/20; 7/19/20)	New	Eastern Europe	CS and SS
13	3 Interviews (6/17/20; 6/18/20; 6/26/20), Statute	New	Eastern Europe	SS
14	2 Interviews (6/16/20; 6/24/20), Statute	New	West Africa	SS
15	3 Interviews (7/24/20; 7/27/20; 7/29/20), Statute	New	West Africa	SS and A

*A* Advocacy (e.g., campaigns for immigrants’ rights), *SS* Social service (e.g., language courses, legal and health assistance), *CS* cultural service (e.g., preservation of cultural heritage and traditions though arts)

### Instruments and Data Collection

Interviews, document analysis and observation were used. The interview protocol was used with 29 leaders and members of MCBOs and included questions on the sociopolitical climate around immigration in the host country (e.g., What is the social and political climate around immigration nowadays in Italy?); organizational changes over time (e.g., Which changes has your organization gone through during these years?); organizational successes (e.g., Which achievements did your organization reach?); and failures (e.g., Were there some things that you could not do?). We also added a specific question about the impact of COVID-19. In addition, ten statutes of MCBOs—that highlight the main objectives and organizational structure of MCBOs—were provided by leaders and became part of the data corpus together with fieldnotes from unstructured participant observation taken during four public events run by three MCBOs between October and December 2019.

All participants signed an informed consent form that explained the study aims and procedure and laid out their right to anonymity at all times. The research protocol was approved by the Ethical Institutional Board of the Catholic University of Milan. Participants did not receive any financial reward, but their participation in the study was promoted on a Web site—that was created for the research—in order to give MCBOs more visibility.

### Procedure and Data Analysis

The research team was made up of five people. Three researchers acted as supervisors, helping build the dataset of the MCBOs, draft the interview protocol and organize frequent debriefing sessions. The other two researchers acted as field researchers and got familiar with the culture of the MCBOs by attending some events, browsing Facebook pages and Web sites (if available), committing time to explain the aims of the research to leaders and building trustworthy relationships with them over a four-month period. Between January and July 2020, the two field researchers interviewed leaders and members in Italian, Spanish or English according to participants’ preferences. As the outbreak of the pandemic in Italy took place in March 2020, and Lombardy—the region where Milan is—was the most affected region, data collection was moved online.

Data analysis started upon collection of data, as recommended in Situational Analysis (Clarke et al., 2018). The data corpus was composed of interviews transcripts, statutes and fieldnotes of observations. The field researchers first engaged separately in open coding and then, together, grouped the codes into categories. Based on the insights provided by participants and using a liberation psychology approach to investigate MCBOs (Paloma & Manzano-Arrondo, 2011), we decided to organize challenges considering three main levels where MCBOs played their mediating role, namely the internal level—i.e., difficulties within MCBOs—the inter-organizational level—i.e., difficulties among MCBOs—and the community level—i.e., difficulties within host societies. We also explored coping strategies and how these challenges were perceived according to some organization-specific markers. For the latter aspect, we took inspiration from Clarke’s positional map (2005)—which is one of the possible maps elaborated through the Situational Analysis. Positional map is an illustration that aims to represent all the major positions articulated in data, operating as “a politics of the acknowledgment of presence instead of denial and repression of diversity” (Clarke et al., 2018, p. 174). Leaders of the 15 MCBOs involved in this study were invited to attend two online meetings to share preliminary results and interpretations, ensure credibility and promote collaboration between the MCBOs and the researchers. In line with this, the findings showed below are the result of these meetings where the ideas from the leaders were integrated with the analysis of the authors.

## Results

In this section, we will present the challenges identified by MCBOs at three levels (internal, inter-organizational and community levels) along with the coping strategies that MCBOs use to maintain their mediating role in the promotion of social justice (see Table 2).

**Table 2:** Challenges and coping strategies among MCBOs

Level	Challenges experienced by MCBOs	Coping strategy used by MCBOs
Internal	Having access to resources	Leadership
	Having engaged long-term immigrant volunteers	
Inter-organizational	Managing competition among MCBOs	International networking
	Overcoming fragmentation among MCBOs due to different sociopolitical narratives	
Community	Overcoming oppressive policies and attitudes	Collective social justice orientation
	Facing manipulation by the host society	

We will then describe the distribution of MCBOs’ challenges according to three organization-specific markers.

### Challenges at Internal Level: Surviving

At this level of analysis, surviving is the main challenge due to the struggle of: (a) having access to resources and (b) having engaged long-term immigrant volunteers.

As for resources, MCBOs said they lacked internal competent staff to seize available opportunities and had limited access to funding that would allow them to hire qualified professionals to design and implement projects: *“We are not sufficiently skilled to be able to apply (…) for funding that can help us stay afloat”* (Leader, MCBO 12).

*You have to find the human resources who can conduct the best projects […] Finding qualified human resources is a challenge we try to overcome every day* (Leader, MCBO 5).

According to our field notes, participants struggle understanding the mechanics of Italian bureaucracy, filling out forms and submitting projects in proper Italian. These struggles also mean that MCBOs find it hard to obtain a venue for their activities from municipalities: “*We have the problem that we don’t have the space, we don’t have money. Meeting at the park is uncomfortable”* (Leader, MCBO 11).

*We have tried several times to find a space. The biggest difficulty is paying the rent. We have had difficulties in finding a space that were, I won’t say “for free” because nothing is free, but at least at a low cost. Even that is difficult!* (Leader, MCBO 5).

For this reason, only a few interviews were implemented in the venues of MCBOs, while most of the organizations’ events that were observed took place in outdoor spaces, in places rented from other organizations or during city events.

As for engagement, MCBOs struggle to incorporate and maintain new volunteers, especially immigrants, because of their living conditions: “*There is also the language problem because many of our compatriots have not learned Italian well enough to be able to express themselves”* (Member, MCBO11); “*[immigrant people] came to the organization to solve some concrete, urgent problems, so many of them, after taking the residence permit, got married, had children, had their own quiet life [and left]”* (Leader, MCBO 4). Another reason is the mistrust that immigrants developed among themselves: “*There is always someone who thinks you are making money through this organization and starts sending [negative] messages on WhatsApp*” (Member, MCBO 7). This mistrust is also connected to some cultural and historical motivations as explained by one interviewee:

*We don’t know what associationism is [...] My compatriots are not united as a community, this is because we are not used to migrating [...] we don’t have this knowledge of how it works. Right now, we are trying to create community through the MCBO, to understand that alone we don’t do anything”* (Leader, MCBO 7).

Moreover, MCBOs find it hard to enhance migrant participation as immigrants need to shift their position from passive recipients (being helped) to active agents (helping others):

*Someone comes to the office because she/he needs something immediately, [for example] some information for the residence permit. [It is difficult for him/her to] understand that those at the office are people who commit their time in a voluntary way to help him/her. So, when you say “you could also do this yourself” they say “No, why should I deal with this?”* (Leader, MCBO 4).

Once immigrants decide to participate, the emotional distress that may result from working with immigrants in need can lead to burnout: “*[Being a volunteer] is a difficult task because sometimes people don’t understand that you can’t solve all their problems, they come here thinking that you should give them everything”* (Member, MCBO 7). Keeping motivation as well as consistency and proactivity is difficult: “*Sometimes you wonder why you should go on, why you should do something when others don’t”* (Leader, MCBO 5).

At this level, relying on strong leadership is the main coping strategy the MCBOs exhibit to face these challenges. Indeed, leaders’ ubiquity and their personal connections are crucial to achieve results:

*“The president is very good at finding professionals who are helpful, for example, she knows a number of opera singers, artists, and she has this divine gift of pleasing people. The biggest strength right now is her charisma”* (Member, MCBO 11).

This is why we observed that the main focus of two out of three events was on the leaders rather than their organization or volunteers. Some MCBOs can also count on the social recognition of leaders who are appreciated for their work and receive practical support (such as spaces, advertisement, funds) from institutions: *“The Consul of our country also helps us a lot, the Consul provides us with a room if we need to hold a big meeting”* (Leader, MCBO 9). The consequence of this dynamic is that work of MCBOs is too leader dependent and weakens the role of the group.

### Challenges at Inter-Organizational Level: Collaborating

This level concerns the hardships in collaborating among MCBOs. It is characterized by two main aspects: (a) managing competition among MCBOs and (b) overcoming fragmentation among MCBOs due to different sociopolitical narratives.

Competition refers to volunteers, projects and festivals as *“Social capital is not that large […] [and therefore] each person is a treasure and every organization tries to get them”* (Member, MCBO 11).

*We organize the “blouse festival”, it is our thing, alright? Okay, one year we organized it [but I told the president that] this didn’t mean anything and the president told me “Why not? We have been organizing it for years! How does [another association] dare [organizing it]?”* (Leader, MCBO 3).

Competition risks corroding relationships among MCBOs that, faced with a perceived scarcity of resources, struggle to see the benefits of coming together. This is why they reported also having troubles organizing migrant coalitions and networks: *“At the beginning we tried to involve a network of organizations, to collaborate with them. Then, we realized that organizations do not understand what it means to run a system, to be part of a network”* (Leader, MCBO 2). As a result, events are usually organized independently by each MCBO, with national organizations or with institutions from countries of origin. Part of this may also be due to the different sociopolitical narratives that characterized MCBOs.

We identified three different narratives among the MCBOs interviewed: blaming migrants, non-alteration and barbarization. The narrative blaming migrants was based on the idea that “Italy cannot welcome everyone” and must establish criteria of access (e.g., immigrants can be accepted as long as they are refugees):

*Some ethnic groups behave according to the law and others don’t, so I agree with stopping illegal immigrants...the latest migration law has some pros and cons. Italy is not a rubber ball that can expand, crime has increased, [life] is more dangerous* (Leader, MCBO 11).

Some participants describe immigrants as “those people on board boats” and tend to disidentify from this group of people. This is especially the case of participants from Eastern Europe who do not want to be called “immigrants” and express an otherness toward “them”. They report that when they arrived, they had to survive alone and show some resentment toward the help that immigrants are now receiving from the Italians: “*In my opinion, much more attention is paid to non-EU immigrants than to EU ones, perhaps for them there are more social projects for integration, for us, there is almost nothing”* (Member, MCBO 13).

According to the non-alteration narrative, nothing has changed in Italy in recent years other than the negative focus of mass media vis-à-vis migration:

*I don’t feel like saying this is a bad moment (...), let’s say the context is difficult and has always been... Unfortunately, today, we have the same problems as ten, five years ago. The law has not changed* (Leader, MCBO 5).

Finally, according to the barbarization narrative, Italy is described as increasingly closed, aggressive and progressively limiting rights, meaning that the country is becoming more barbaric (i.e., cruel) and it is no longer important to “conceal” racism as it has become a new socially accepted norm: *“Italian people look down on us because they are ignorant, they do not know who we are, they watch the TV where everyone says ‘he has disembarked’ but I have not disembarked”* (Leader, MCBO 15). MCBOs embracing this narrative advocate for immigrants’ rights to freely circulate and show a strong critical thinking on the historical system of power: *“We are returning to the situations that existed in the past […] the amount of people who died in the boats due to the slave trade is very high… wealth was built on that”* (Leader, MCBO 4).

Having different narratives may cause MCBOs to not recognize one another as having the same purpose while being externally perceived as not cohesive and without a unified voice when requesting better conditions for immigrants in the host country. We were able to observe this also in the feedback meeting we conducted with the leaders on the preliminary results, where participants were not very willing to identify common struggles among them and somewhat maintained their stance and narratives.

To face such challenges, MCBOs try to build networks not at local but at international level based on their origin or political position:

*“Our network is worldwide. The most important thing that we have is the network because there are some [people] in the United States, some in France and Germany, because we are everywhere [...] so we have this network, which is very important”* (Leader, MGO 7).

### Challenges at Community Level: Being Recognized as Mediating Actors

This level refers to MCBOs’ struggle to build a reputation as mediating actors within the wider community. In particular, this challenge was related to two aspects: (a) overcoming oppressive policies and attitudes and (b) facing manipulation by the host society.

The first aspect refers to migration policies and attitudes among Italian people that make the work of MCBOs harder but also more needed. Indeed, according to the most critical participants, MCBOs try to fight pervasive indifference, non-recognition of migrants as human beings, repatriations and increasing barriers that characterize the daily life of immigrants:

*Milan has gotten worse [...] For example, think about concerts: they didn’t give any chance to African artists [to perform]. Have you ever seen an immigrant singing at Radio Italia concerts in Duomo? The Milanese people don’t even care about foreigners […] they didn’t give us the space to live, because for them we are animals* (Leader, MCBO 15).

Faced with this perceived context, MCBOs feel the need to carve out spaces for their action.

The second aspect refers to manipulation, as MCBOs often feel exploited by national organizations:

A*nother very sensitive point [...] is the exploitation—often for their own benefit—of migrant-structured entities such as organizations, artistic groups and so on. It feels like it is nice [that these organizations] exist only as long as there is a need for them* (Leader, MCBO 6).

This is the reason why an interviewee reports that: “*[what we want is] get at the same level. This is the work that I do and we also want to do as an organization, because otherwise we won’t get anywhere”* (Leader, MCBO 3). MCBOs are aware that it is important to maintain relations with national organizations. For example, during the events we attended, we observed that representatives of host institutions had been invited. Nevertheless, the challenge is to do so within a symmetric power structure.

Faced with such challenges, some MCBOs reported a strong collective social justice orientation as their main coping strategy: “*I see the fighting spirit, that desire to go further, not limiting yourself to do only that specific thing”* (Leader, MCBO 4).

### Challenges According to some Organization-Specific Markers

**Fig. 1:**
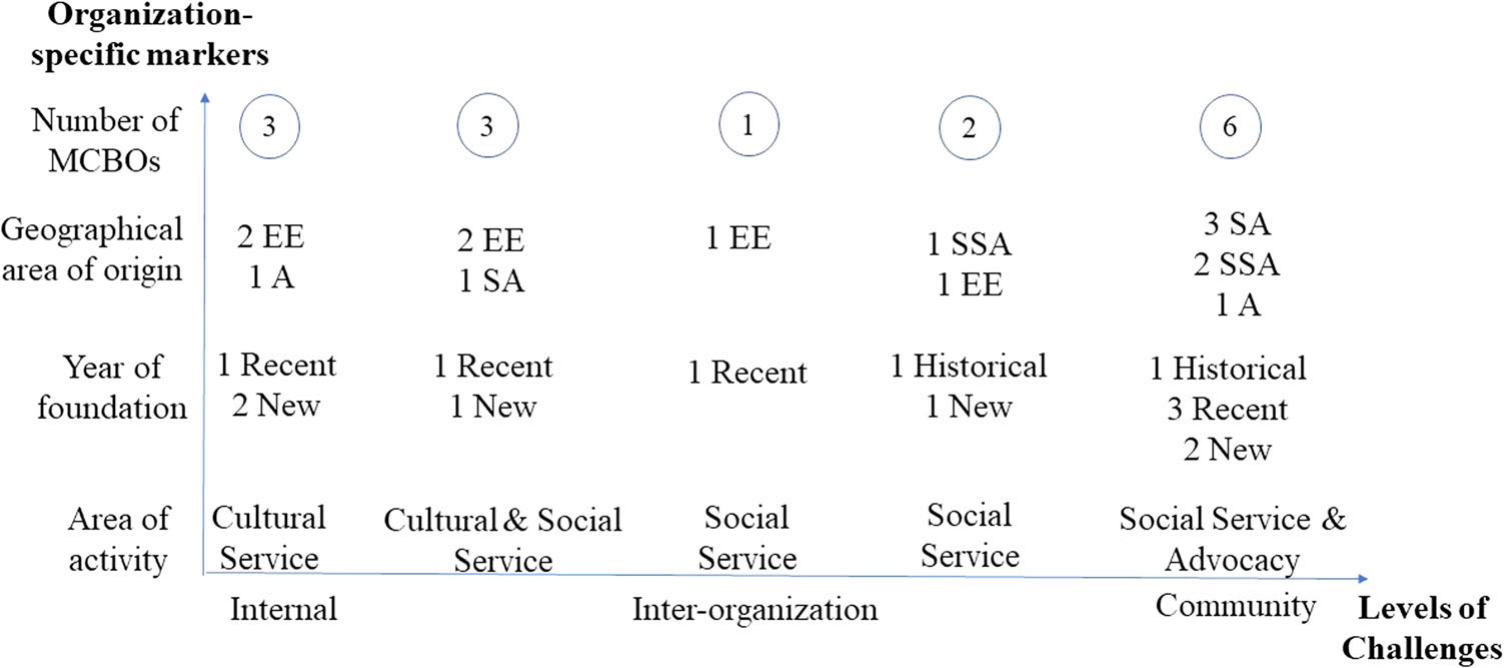
Positioning of the challenges faced by MCBOs at the three levels considering organization-specific markers. *A* Asia, *EE* Eastern Europe, *SA* South America, *SSA* Sub-Saharan Africa

To answer the second research aim, we focused on three organization-specific markers, i.e., geographical area of origin, year of activities and area of activity. Figure 1 shows the distribution of MCBOs according to the type of challenges experienced and indicates the main trends.

As for the geographical area of members of MCBOs, we found that for most of the South American and Sub-Saharan African organizations challenges are present at the community level. Eastern European MCBOs more frequently face challenges at the inter-organizational and internal levels and Asian MCBOs positioned themselves between the internal and community level. Considering the year of activity of the MCBOs, our results suggest that historical MCBOs—founded before 2000—perceived that their problems were linked mainly to the community context in which they operated. By contrast, recent MCBOs—founded after 2000—and new MCBOs—founded after 2010—featured more fragmentation. We did find some patterns that help explain the different positioning of MCBOs, also considering the area of their activities. The results suggest that the MCBOs focusing on cultural services (e.g., remembering the country of origin, spreading cultural knowledge) perceived challenges at lower levels, whereas introducing social services (e.g., helping and supporting immigrants) led MCBOs to perceive more structural challenges. Finally, when helping was paired with mediation activities between immigrants and national institutions and with fight for immigrant rights (i.e., advocacy), the MCBOs usually experienced challenges at community level.

## Discussion

This paper argues that MCBOs are mediating structures between immigrants and the host society that facilitate intercultural relations and provide the opportunity to promote a liberation process and pursue social justice (Paloma et al., 2010; Todd & Allen, 2010). The results of this study highlight six main challenges and the corresponding coping strategies at three different levels: (a) internal level (i.e., having access to resources and having engaged long-term volunteers, both challenges addressed through strong leadership); (b) inter-organizational level (i.e., managing competition among MCBOs and overcoming the fragmentation among MCBOs due to different sociopolitical narratives, both challenges addressed through international networking); and (c) community level (i.e., overcoming oppressive policies and attitudes and facing manipulation by the host society, both challenges addressed through strong collective social justice orientation). To tackle these challenges, MCBOs should act synergically with academics, policymakers and community stakeholders in host societies. We suggest some lines of action that can be directly promoted by MCBOs in collaboration with these actors.

The challenges identified at the intra-organization level are in line with the results by Papadopoulos et al. (2013) and Gleeson and Bloermraad (2012) and indicate that MCBOs have low level of social capital, resources and opportunities. We found that many MCBOs find attracting and maintaining volunteers hard and experience a lack of qualified professionals. Hout (2013) also reported that because funds are usually assigned to mainstream organizations, MCBOs struggle being selected under calls for proposals. Moreover, as highlighted by Kyrieri and Brasser (2012), MCBOs have high levels of instability in their activities. We found this may be particularly due to different reasons, i.e., hardship in handling Italian bureaucracy, historical and cultural differences in the way immigrant volunteers engage within MCBOs, and emotional distress resulting from the work that MCBOs conduct. The hardships in recruiting and maintaining volunteers may decrease the external impact of any community-based organizations, as suggested by Maton (2008), but is even more present in the case of MCBOs due to structural oppression. Moreover, it carries the risk of strongly relying only on the leaders. As argued by Ortiz (1981), the retention of power and autonomy by the leadership of organizations may supersede the objective of promoting social justice.

In order to overcome these challenges, we suggest two main lines of action: (a) helping MCBOs obtain funding to hire qualified people and (b) promoting the image of MCBOs to attract volunteers, providing psychological support and training on leadership and management to avoid burnout and sustain volunteers’ motivation. These actions would increase the social capital of MCBOs, would underpin specific recruitment campaigns to present MCBOs’ activities to new potential volunteers—also by building Web sites—and, more importantly, would expand leadership beyond individuals and increase the potential of promoting social justice within these settings.

At the inter-organizational level, we found that creating connections *among* MCBOs is a challenge. Indeed, MCBOs experience competition due to the impossibility to perceive common interests and the different narratives they assume on the Italian sociopolitical context. Indeed, some MCBOs aligned with the mainstream narrative of blaming migrants, while others were more critical and felt a climate of “barbarization”. These differences may be responsible for the hardship in organizing joint advocacy activities in the local community. Gigliotto et al. (2022) argued that “while collaboration could be fruitful, due to limited resources or administrative challenges there may be tensions arising among groups that struggle for representation in the institutional heritage discourse” (p. 88). Martinez-Damia et al. (2021) also found that competition can be a barrier to attracting new immigrant volunteers, who may not know how to choose which MCBO will best fit their needs.

To support MCBOs overcome these challenges, we suggest implementing two lines of action: (a) encouraging collaboration between MCBOs for mutual support vis-à-vis common challenges and (b) stressing the social value of diversity within the immigrant community and designing group training on critical thinking and liberation strategies among MCBOs. These actions refer to the importance of building collaborative capacity, i.e., the conditions needed by organizations to work together toward shared goals in order to create sustainable community changes (Goodman et al., 1998). Garcia-Ramirez et al. (2009) described how teams composed of academics can obtain successful results when implementing programs addressed to immigrants by building common resources and a sense of collective efficacy. While doing so, MCBOs should keep in mind that the sense of human commonality and the collective desire to change specific situations is the most important factor (Lai, 2010).

Regarding the challenges identified at community level, we highlighted that MCBOs felt the need to confront oppression stemming from migration policies and attitudes among Italian people and confirmed to perceive manipulation by national organizations as previously reported (Gigliotto et al., 2022; Kyrieri & Basser, 2012). Paloma and Manzano-Arrondo (2011) referred to the latter phenomena also as tokenism and suggested that its main risk is to perpetuate the status quo. It seems that MCBOs know they are being used but do not know how to get out of this logic. Pilati (2012) highlighted that MCBOs often struggle to mobilize because “in the Italian context, where the new multi-ethnic landscape is hardly recognized, the institutions seem to legitimate more the role of Italian organizations in the field of immigration” (p. 684).

To face these challenges, we suggest two main lines of action: (a) rising awareness on oppressive power dynamics and helping MCBOs find strategies in order to avoid tokenism and (b) helping increase the prestige of MCBOs through agreements with national institutions. In this perspective, some seminars promoting respect, diversity and cultural humility among MCBOs and national organizations could be implemented. Partnerships with universities could be useful to amplify the voice of MCBOs and create opportunities where they can be the ones launching initiatives and taking decisions.

Finally, some organization-specific markers relate to these challenges. MCBOs that carried out mainly social and cultural services tended to perceive more challenges at the internal level, while in cases where political activities were also implemented, they perceived more challenges at the community level. Unsurprisingly, we can imagine that MCBOs that are more engaged in advocacy have members with stronger critical thinking and frame their struggle at structural level. The literature (Mellinger, 2014) reports that organizations created to provide social support usually continue to prioritize this mission and engage less in political activity due to limited funding, misunderstanding of legal regulations and misperceptions of what constitutes advocacy. Furthermore, MCBOs composed of people from South America and Sub-Saharan Africa seemed to perceive challenges at community level, while those gathering people from Eastern Europe seemed to perceive challenges at internal level. The heterogeneity of the positions helps avoid a stereotyped vision of MCBOs as a single entity and recognize intergroup diversity according to the degree of privileges and critical thinking on the sociopolitical system in which they are involved.

This study is not without limitations. Firstly, as the MCBOs included are limited to Northern Italy, it is difficult to apply the findings to other MCBOs in other host countries. Moreover, the city of Milan can be considered as more “welcoming” compared to the rest of Italy, so future comparative research should be conducted not only in other countries but also within Italy. Secondly, because data collection strongly relies on in-depth interviews, challenges MCBOs may face are “top-down” and potentially biased by social desirability. Other methods (e.g., informal meetings, participant observations, analysis of statues) were employed to partly address this issue. Nevertheless, the relatively low number of ethnographic observations conducted due to the COVID-19 pandemic has to be noted. Thirdly, in this study we only included MCBOs that were still active in the city as it was very hard to engage with those that were not or only performed a few activities. We therefore may have missed some important challenges experienced by these MCBOs. Moreover, starting our recruitment from an institutional network like the ACM may have narrowed the selection of MCBOs engaged to the more “institutionalized” ones. We tried to overcome this issue by contacting new MCBOs who were not part of ACM in the first place.

## Conclusion

MCBOs are mediating structures as they act between the structural and individual level in order to bring social justice and individual well-being through a collective process of liberation. MCBOs will be successful in their mediating role only if the challenges they currently face are recognized and overcome. The present study enriches the literature by focusing on the challenges faced by MCBOs as mediating structures within host societies. Building on the identified challenges and coping strategies, the study provides guidelines for actions that MCBOs, together with academics, policymakers and community stakeholders, can implement to promote social justice for all groups. We proved that MCBOs may have unique experiences based on the country of origin of their members, so future research may further explore the diversity and specificity of challenges faced by MCBOs based on their cultural background.
